# Effect of Positive Thinking Skills on Optimism and Death Anxiety of COVID-19 Nurses: A quasi-experimental study

**DOI:** 10.4314/ejhs.v34i2.5

**Published:** 2024-03

**Authors:** Mohammadreza Doustinouri, Mozhgan Rahnama, Abdolghani Abdollahimohammad, Mahdieh Poodineh Moghadam, Elham Shahraki Moghadam, Elaheh Asadi-Bidmeshki

**Affiliations:** 1 Student Research Committee, School of Nursing and Midwifery, Zabol University of Medical Science, Zabol, Iran; 2 Department of Nursing, School of Nursing and Midwifery, Zabol University of Medical Sciences, Zabol, Iran

**Keywords:** Optimism, Anxiety, Attitude to Death, COVID-19, Nurses

## Abstract

**Background:**

The COVID-19 pandemic has placed nurses on the front lines, facing challenging conditions and increased stress levels. The aim of this study was to examine the effect of positive thinking skills training on nurses' optimism and death anxiety while caring for COVID-19 patients.

**Methods:**

In a quasi-experimental, two-group, pre- and post-test study, a total of 52 eligible nurses working at Amir al-Momenin Hospital in Zabol, Iran, between October and December 2022, were divided into intervention and control groups. The intervention group received positive thinking skills training via email or WhatsApp, with eight, 60-minutes sessions over two months. Data were collected using a demographic questionnaire, the Templer Death Anxiety Scale (DAS), and the Scheier and Carver Life Orientation Test, both before and one month after the intervention.

**Results:**

There were no significant differences in optimism and death anxiety scores between the two groups before the intervention. However, the intervention group showed a statistically significant increase in optimism (from 13.11±3.78 to 19.03±2.58) and decrease in death anxiety (from 55.15±7.06 to 41.76±4.48) after the intervention (P <0.001).

**Conclusion:**

Considering the stressful nature of COVID-19 disease, positive thinking skills training can be recommended as an effective method to reduce death anxiety and improve nurses' optimism, thus enhancing their willingness to continue caring for patients.

## Introduction

The coronavirus disease-2019 (COVID-19) pandemic began in Wuhan, China, and spread rapidly around the world, causing a significant number of deaths (1), including among healthcare providers, especially nurses (2-3). Nursing is a stressful profession with a high level of anxiety (4), and the COVID-19 pandemic has further increased nurses' death anxiety (5). Compared with other healthcare professionals, nurses tend to experience higher levels of death anxiety (6). Death anxiety is a complex concept that generally refers to the fear of one's own death and the death of others (7). This anxiety affects nurses' mental health (4) and the quality of care they provide, especially to dying patients (4, 8). Interestingly, personal characteristics such as optimism are positively associated with death anxiety (8). Therefore, it is critically important to increase the level of optimism among nurses, especially as they are at the forefront of the fight against the COVID-19 pandemic (4).

One of the most effective ways to improve knowledge and attitudes and instill positive thinking for better psychological well-being is through training (9). Studies have shown a significant positive impact of positive thinking training on nurses' engagement, resilience, job burnout (10), and happiness (9). Therefore, it is imperative to improve nurses' optimism and reduce their death anxiety through a low-cost strategy such as an empowerment training program. The aim of this study was to determine the effect of positive thinking skills on the optimism and death anxiety in nurses caring for COVID-19 patients. We hypothesized that positive thinking skills training could improve optimism and reduce death anxiety in nurses caring for COVID-19 patients.

## Methods

This quasi-experimental, two-group, pre- and post-test study was conducted among nurses experienced in caring for COVID-19 patients at Amir al-Momenin Hospital in Zabol, Iran. The study was conducted between October and December 2022.

The inclusion criteria were as follows: willingness to participate in the study, having at least a bachelor's degree in nursing, no history of positive thinking skills training, at least 6 months of experience in caring for COVID-19 patients, no known mental disorders (especially obsessive-compulsive disorder, generalized anxiety disorder, or phobias), and no concurrent participation in similar interventions. On the other hand, the exclusion criteria included unwillingness to cooperate at any stage of the study, incomplete filling of the questionnaire, absence from training sessions, and divorce or death of a relative during the study. A total of 52 eligible nurses were randomly assigned to the intervention (n=26) and control (n=26) groups.

After obtaining nurses' consent to participate, data were collected using three questionnaires. The first questionnaire collected demographic information including age, gender, marital status, education level, years of work experience, and experience in caring for COVID-19 patients. The second questionnaire was Scheier and Carver's Life Orientation Test (optimism), which is a 5-point Likert scale ranging from 0 to 4, consisting of 10 questions (11). Its reliability was confirmed in previous studies (12-13). The third questionnaire was Templer's Death Anxiety Scale, a self-administered questionnaire with 15 five-point Likert-type items (14). The validity and reliability of this scale were confirmed in a previous study (15). Cronbach's alpha for Scheier and Carver's Life Orientation Test and Templer's Death Anxiety Scale were calculated to be 0.70 and 0.81, respectively.

Before the educational content was delivered, the intervention group was divided into five groups and coded from 1 to 5. The content was delivered to group 1 on Saturday and to group 5 on Wednesday. On the 6th day, the researcher and the participants discussed the content individually. The intervention program consisted of eight 60-minute sessions, over 2 months, with text and audio content delivered via email or WhatsApp with 5-day intervals between sessions. Participants were strictly prohibited from sharing the content until the end of the study.

The educational content included topics such as positive thinking, recognizing positive thinking symptoms, analyzing one's perspective, techniques to reduce death anxiety, using informative language and words, maintaining positive behavior, self-esteem, building optimism, and self-confidence. Other topics included controlling emotions (avoiding guilt, managing anger, coping with anxiety, and overcoming jealousy), self-confidence techniques, focusing on strengths rather than weaknesses, basic steps for self-expression, assertiveness (saying no), managing life events, creating a positive environment, maintaining positive health, fostering positive relationships with others, and coping with everyday life problems. The control group received no training during the study, but did receive positive thinking training after the study was completed..

The nurses were asked to complete the questionnaires again one month after the intervention. Meanwhile, the control group, which did not receive any training, also completed the questionnaires again one month after the pretest.

**Sample size calculation**: The sample size was calculated based on the study by Kyani et al. (9). Using a type I error of 5% and a study power of 95%, Cochran's sample size calculation formula indicated that 52 eligible subjects would be needed for this study.

**Statistical analysis**: All analyses of quantitative data were performed using the Statistical Package for the Social Sciences (SPSS) 23.0. The Shapiro-Wilk test was used to determine whether the data obtained from the groups were normally distributed. Descriptive statistics were used to summarize the characteristics of the study participants. The independent t-test, Mann-Whitney U test, and Chi-squared test were used to evaluate the difference of variables between the intervention and control groups. A P-value less than 0.05 was considered statistically significant.

**Ethical considerations**: The study was conducted in accordance with the Declaration of Helsinki. This study was approved by the ethics committee of Zabol University of Medical Sciences, Zabol, Iran (code: IR.ZBMU.REC.1401.065). The participants were informed about the purpose of the study and the confidentiality of the data, and informed consent was obtained from all of them.

**Data sharing**: All relevant data and methodological details of this study will be made available to interested researchers upon reasonable request to the corresponding author.

## Results

A total of 52 nurses caring for COVID-19 patients participated in this study. The results showed that the mean scores for age of the nurses in the intervention and control groups were 33.53 (range: 25-50) and 36.57 (range: 27-50) years, respectively. The mean years of work experience of the intervention and control groups were 12.03 (range: 2-23) and 9.83 (range: 3-21) years, and the mean work experience in the COVID-19 unit was 4.53 (range: 2-6) and 4.46 (range: 2-6) months, respectively.

The independent t-test showed no significant difference between the intervention and control groups in terms of age, years of work experience, and work experience in the COVID-19 unit (P<0.05). In addition, the Mann-Whitney U test showed no significant difference between the intervention and control groups in terms of work experience in the COVID-19 unit (P<0.05). The chi-squared test showed no significant difference between the intervention and control groups in terms of gender, marital status, and experience of death among close relatives or friends (P<0.05).

However, there was a significant statistical difference between the two groups in terms of educational level (P=0.017) (Table 1). Table 2 shows that there was no significant difference in the mean scores of optimisms and death anxiety between the two groups. However, there was a significant difference in the mean scores of optimisms and death anxiety between the intervention and control groups (P<0.001).

**Table 1:** Individual characteristics of nurses in intervention and control groups

Variable		Intervention	Control	*P-value*
**Gender**	Male	12(46.2)	9(34.6)	0.397
**N (%)**	Female	14(53.8)	17(65.4)	
**Marital status**	Married	20(76.9)	20(76.9)	1
**N (%)**	Single	6(23.1)	6(23.1)	
**Age (year)**	*Mean (SD)*	33.53 (6.35)	36.57 (6.32)	0.09
**Job experience (year)**	*Mean (SD)*	12.03 (5.14)	9.88 (5.45)	0.149
**Work experience in the COVID-19 ward (Month)**	*Mean* (SD)	4.53 (1.17)	4.46 (1.39)	0.830

**Table 2:** Comparison of the mean and standard deviation of optimism and death anxiety scores in intervention and control groups before and after the intervention

Variable		Mean (SD)		Score	95% CI	P-value

		Intervention	Control			
**Optimism**	Before	13.11 (3.78)	12.38 (3.55)	-0.71	(-2.77,1.31)	0.477
	After	19.03 (2.58)	13.38 (3.22)	-6.97	(-7.28, -4.02)	<0.001
**Death anxiety**	Before	55.15 (7.06)	56.34 (8.42)	0.55	(-3.13,5.52)	0.583
	After	41.76 (4.48)	55.15 (8.25)	7.26	(9.68,17.08)	<0.001

## Discussion

The study found high pre-intervention death anxiety scores among nurses caring for COVID-19 patients in both the intervention and control groups. Previous studies also reported a high prevalence of death anxiety among nurses (6-7, 16).

In addition, before the intervention, both groups of nurses showed moderate levels of optimism, which is consistent with a study of nursing students (17). The findings highlight the need for interventions to reduce death anxiety and increase optimism in nurses. The intervention, which included training in positive thinking skills, was effective in reducing death anxiety and improving nurses' optimism. Optimism has been found to be positively correlated with self-control (12) and work performance (18), and it also influenced adaptation to life events and problem-solving behaviors (17). The improved optimism of the nurses in this study may have been due to a sense of security that developed after the COVID-19 pandemic following the program. Other studies have also supported the positive effects of positive psychotherapy techniques on reducing anxiety in nurses (19) and the negative association between fear of COVID-19 and positive thinking (20). Optimism has been identified as a factor that positively affects the mental health of nurses working in COVID-19 units (21) and is associated with good general health (22). Positive psychology techniques have also been found to be effective in changing the beliefs of nursing students (23). Therefore, the improved optimism scores in the current study could be attributed to a change in the nurses' beliefs. In a study by Motamed-Jahromi et al, positive thinking training using a social media application was shown to improve nurses' quality of work life (24). The results of another study showed that nurses' levels of happiness and resilience were improved by the teaching of positive thinking skills (25).

According to the researchers, the reduction in death anxiety after positive thinking skills training in the present study may be due, in part, to the improvement in the nurses' mental health. Previous research showed a significant relationship between death anxiety and mental health in nurses (6). Other studies also highlighted the importance of nurses' mental health and the need for authorities to pay attention to this aspect (7). Results from a study of nurses in Korea showed the effect of a death preparation education program on reducing death anxiety and a positive effect on nurses' attitudes toward death and end-of-life care (26).

Positive thinking skills training has been associated with several positive effects on nurses' mental states, including increased happiness, decreased job burnout, and improved job satisfaction (9,10). Gratitude, one of the positive thinking skills taught in the training, has been associated with increased happiness and hope (27). In addition, the study suggests that the nurses' improved optimism after the positive thinking skills training contributed to the reduction in death anxiety. Optimism has been identified in previous research as a predictor of death anxiety in nurses (8).

The use of audio files as a positive thinking skills training tool in the present study also had a positive effect. To confirm this training method, Norouzi et al. suggest that teachers need to create and propose new methods to improve their training skills (28). However, the researchers acknowledge the limitation of not being able to control the training that nurses received from other sources (e.g., books, television) during the intervention.

There are some limitations of the present study that need to be addressed. Our study is a single-center study, and selection bias is possible, which necessitates conducting similar studies in other settings, countries, etc. Another limitation of the study was the collection of post-test data one month after the completion of the training program, which did not show a long-term effect. We recommend that similar studies collect post-test data after several months. In addition, the positive thinking skills training was delivered via email or WhatsApp, which may limit the interactive and personalized nature of the intervention. Differences in individual engagement with the intervention or the level of support provided by the researchers may have influenced the results.

In conclusion, according to the results of this study, nurses caring for COVID-19 patients had high death anxiety but low optimism scores. The intervention, positive thinking skills training, was found to reduce their death anxiety and increase their optimism. This training helped the nurses gain a sense of control over critical situations, improve their ability to cope with problems, reduce anxiety, and improve their mental health. Considering the stressful conditions of the COVID-19 pandemic for nurses and the shortage of human resources in the field, positive thinking skills training is recommended as a simple and effective method to reduce death anxiety and increase optimism, thus encouraging nurses to continue providing care.

## References

[1] Raoofi A, Takian A, Akbari Sari A, Olyaeemanesh A, Haghighi H, Aarabi M (2020). COVID-19 Pandemic and Comparative Health Policy Learning in Iran. Arch Iran Med.

[2] Emami Zeydi A, Ghazanfari MJ, Sanandaj FS, Panahi R, Mortazavi H, Karimifar K, Karkhah S, Osuji J (2021). Coronavirus Disease 2019 (COVID-19): A Literature Review from a Nursing Perspective. Biomedicine (Taipei).

[3] Ghazanfari MJ, Esmaeili S, Emami Zeydi A, Karkhah S (2022). Moral distress among nurses during COVID-19 pandemic: Challenges and coping strategies. Nurs Open.

[4] Siam BGAH, ALreshidi OAS (2023). Emergency Nurses' Compliance with Standard Precautions during the COVID-19 Pandemic at Governmental Hospitals in Hail City, Kingdom of Saudi Arabia. Ethiop J Health Sci.

[5] Sanadgol S, Firouzkouhi M, Badakhsh M, Abdollahimohammad A, Shahraki-vahed A (2020). Effect of guided imagery training on death anxiety of nurses at COVID-19 intensive care units: a quasi-experimental study. Neuropsychiatria i Neuropsychologia.

[6] Iddrisu M, Poku CA, Mensah E, Attafuah PYA, Dzansi G, Adjorlolo S (2023). Work-related psychosocial challenges and coping strategies among nursing workforce during the COVID-19 pandemic: a scoping review. BMC Nurs.

[7] Nia HS, Lehto RH, Ebadi A, Peyrovi H (2016). Death Anxiety among Nurses and Health Care Professionals: A Review Article. Int J Community Based Nurs Midwifery.

[8] Peters L, Cant R, Payne S, O'Connor M, McDermott F, Hood K, Morphet J, Shimoinaba K (2013). How death anxiety impacts nurses' caring for patients at the end of life: a review of literature. Open Nurs J.

[9] Kyani P, Naeini SMK, Safavi M (2020). The effect of positive thinking training on happiness and resilience of nurses in intensive care units: a randomized clinical trial. Med Sci J Islamic Azad Univ Tehran Med Branch.

[10] Mehafarid M, Khakpour M, Jajarmi M, Alizadeh Mousavi A (2015). Effectiveness of positive thinking training on hardiness & resilience and Job burnout in women nurses. Journal of Nursing Education.

[11] Rahnama N, BakhtiyarPour S, Bavi S, Jayervand H, DashtBozorgi Z (2021). Presentation a Model of Quality of Work Life Based on Job Stress and Optimism Mediated by Fatigue in Married Nurses in Ahvaz. J Health Promot Manag.

[12] Kajbaf M, Oeeyzi HR, Khodabakhshi M (2006). Standardization, reliability, and validity of optimism scale in Esfahan and a survey of relationship between optimism, self mastery and depression. Psychol Stud.

[13] Hinz A, Schulte T, Finck C, Gómez Y, Brähler E, Zenger M, Körner A, Tibubos AN (2022). Psychometric evaluations of the Life Orientation Test-Revised (LOT-R), based on nine samples. Psychol Health.

[14] Zuccala M, Menzies RE, Hunt CJ, Abbott MJ (2022). A systematic review of the psychometric properties of death anxiety self-report measures. Death Stud.

[15] Sharif Nia H, Ebadi A, Lehto RH, Mousavi B, Peyrovi H, Chan YH (2014). Reliability and validity of the persian version of templer death anxiety scale-extended in veterans of iran-iraq warfare. Iran J Psychiatry Behav Sci.

[16] Moudi S, Bijani A, Tayebi M, Habibi S (2017). Relationship between death anxiety and mental health status among nurses in hospitals affiliated to Babol university of medical sciences. J Babol Univ Med Sci.

[17] Khanbabayi Gol M, Mir Mazhari R, Dorosti A (2018). Relationship between procrastination and optimism in undergraduate nursing students of medical sciences universities of Northwest Iran-1397. Journal of Nursing Education.

[18] Luthans KW, Lebsack SA, Lebsack RR (2008). Positivity in healthcare: relation of optimism to performance. J Health Organ Manag.

[19] Navvab Daneshmand M, Sharifi T, Mashhadizadeh S, Ahmadi R (2022). Efficacy of Positive Psychotherapy on the Resilience of Female Nurses with Anxiety Symptoms. Res Behav Sci.

[20] Bakioğlu F, Korkmaz O, Ercan H (2021). Fear of COVID-19 and Positivity: Mediating Role of Intolerance of Uncertainty, Depression, Anxiety, and Stress. Int J Ment Health Addict.

[21] Kheradmand M, Zadafshar S, Abaskhanian Davanloo F, Yazdkhasti F (2021). Role of Optimism of Mental Health and Post-Traumatic Growth in Coronavirus Unit's Nurses By Mediation of Resilience and Cognitive Emotion-Regulation. J Appl Psychol.

[22] Issazadegan A, Sheikhy S (2011). The relationship between optimism and emotion regulation strategies with general health. Nursing & Midwifery Journal.

[23] Yavari Barhaghtalab E, Seirafi M, Kalhorinia Golkar M (2020). Structural relations between physical and mental health based on flourishing mediated by optimism in nursing students. Iran J Nurs.

[24] Motamed-Jahromi M, Fereidouni Z, Dehghan A (2017). Effectiveness of Positive Thinking Training Program on Nurses' Quality of Work Life through Smartphone Applications. Int Sch Res Notices.

[25] Ansarimehr F, Zarea K, Farsi Z, Haghighizadeh MH (2022). The Effect of Positive Thinking Skills Training on Happiness and Resilience of Nurses in Shahid Baqaei Oncology Hospital of Ahvaz. Iran J Psychiatr Nurs.

[26] Chu EY, Jang SH (2021). The Effects of a Death Preparation Education Program on Death Anxiety, Death Attitudes, and Attitudes toward End-of-Life Care among Nurses in Convalescent Hospitals. J Hosp Palliat Care.

[27] Ahmadpoori SF, Mottagi M (2019). The effect of positive thinking skills (optimism) based on the thanksgiving notebook on the spiritual health of adolescent girls. Iran J Nurs Res.

[28] Nourozi H M, Rokhi F, Karimi Moonaghi H (2013). Comparison of Video-Based Instruction and Instructor Demonstration on Learning of Practical Skills in Nursing Students. Iran J Med Edu.

